# BIOLOGICS IN DERMATOLOGIC THERAPY – AN UPDATE

**DOI:** 10.4103/0019-5154.55627

**Published:** 2009

**Authors:** Arijit Coondoo

**Affiliations:** *From the Department of Dermatology, Vivekananda Institute of Medical Science, Kolkata, India.*

**Keywords:** *Biologics*, *interleukin*, *interferon*, *granulocyte macrophage*

## Abstract

Biologics are protein molecules which are used in various diseases to target the specific points in the immunopathogenesis of the diseases. The molecules are produced by recombinant DNA technology. The molecules bind to the specific targets without interfering wtih rest of the pathogenetic pathways. Therefore the so called ‘immunosuppressives’ have, although, a broader broader spectrum of action on immune system, their side-effects are also equally more. The biologics, because of their spefic action on the immune system, have very little side effects. The biologics which have revolutionized the treatment of various dermatologic diseases have been discussed here.

## Introduction

The term biologics, broadly defined, includes a wide range of substances such as vaccines, blood and blood components, allergens, somatic cells, gene therapy, tissues, and recombinant therapeutic proteins.[1] However, biologics are generally used only to refer to protein molecules that are therapeutically used in various diseases to target the various immunological processes in these disorders at specific points on their inflammatory cascade.[2] They are produced by recombinant DNA technology, whereby, copies of the relevant genes that play an important role in a disease process are replicated to produce the specific protein.[3] The new molecules then bind specifically to the target to produce the desired effect, by activating or deactivating the target without interacting with other proteins. They persist in the body for a prolonged period thus preventing the need for frequent dosing and do not induce an immune response themselves, as they are immunologically as ‘silent’ as possible.[4] While ‘traditional’ immunosuppressive drugs have a broader impact on the immune system resulting in a large number of side-effects, biologics block the disease process at a much earlier stage and are more specific in targeting the immune process so that the number of side- effects are usually reduced.[5]

## Classification

Biologics are divided into three groups:[6]

1. Recombinant human cytokines and growth factors
2. Monoclonal antibodies
3. Fusion antibody proteins

## Recombinant human cytokines and growth factors

Cytokines are water soluble non-immunoglobulin proteins and glycoproteins produced by a wide variety of cells in the human body and released transiently into the tissue microenvironment in response to any immune stimulus.[7,8] Recombinant cytokines or cytokine antagonists produced by recombinant DNA technology have been used as immunomodulators for malignant and inflammatory dermatoses.[9] The principal recombinant cytokines used in dermatology are:

- Interferon α (IFNα)
- Interferon γ (IFNγ)
- Interleukin 1 Receptor antagonist (IL1Ra)
- Interleukin 2 (IL-2)
- Interleukin 4 (rhIL-4)
- Interleukin 10 (rhIL-10)
- Interleukin 11 (rhIL-11)
- Granulocyte macrophage colony stimulating factor (GM-CSF)
- Platelet derived growth factor (PDGF)

### Interferon α

Interferons were first described by Isaacs and Lindenmann in 1957.[10] IFNα, a family of about 20 related proteins, is produced by virus-infected leucocytes, in response to an appropriate stimulus. Along with other Type I Interferons (IFNβ, IFNε, IFNκ, and IFNω) it is expressed as a first line of defense against viral infections and limits the viral spread during the initial stages of an infection by directly inhibiting a viral replication in infected cells and indirectly by stimulating the adaptive immune system.[11] It also stimulates the natural killer cell activity, increases the expression of the MHC class I molecules, and promotes production of Th1 cells, while inhibiting the development of Th2 cells. Thus IFNα also exhibits antiproliferative and immunomodulatory functions.[12] Recombinant IFNα is given as a subcutaneous or intramuscular injection to treat verruca vulgaris,[13] condyloma acuminatum,[14] cutaneous T cell lymphoma,[15] Kaposi's sarcoma (AIDS related),[16] melanoma,[17] basal cell carcinoma,[18] squamous cell carcinoma,[19] actinic keratosis,[20] Behçet's disease,[21] hemangiomas[22] and keloids.[23] The injections are usually given thrice weekly and the dose (depending on the condition being treated) varies from low-dose therapy for condyloma acuminatum[24] to high-dose therapy for melanoma.[25] Of late, pegylated IFNα is being used for convenience, because it has a longer half-life (resulting in increased patient tolerability) and hence can be given once weekly.[15] IFNα therapy is associated with a number of adverse effects — the commonest being flu-like symptoms of fever, headache, chills, sweats, myalgia, and arthralgia. Pretreatment with acetaminophen may prevent these symptoms and the symptoms gradually improve with subsequent doses, usually within 10 days of starting the therapy.[26] Injection site reactions such as local redness, pain, and swelling may occur. Potentially dangerous side effects are autoimmune reactions, adverse cardiovascular effects, and depression. The patients may also experience fatigue, dizziness, weight loss, anorexia, photosensitivity, partial alopecia, hypothyroidism, peripheral neuropathy, psychosis, and sexual dysfunction.[15,27]

### Interferon γ

IFNγ is produced by activated Th1 CD4 T cells, CD8 T cells, and NK cells. Although it displays some of the antiviral properties of IFNα, its predominant biological role is to stimulate the adaptive immune system and suppress production of Th2 cytokines. It activates macrophages, enhances expression of the MHC class II antigen and increases the cytotoxic activity of natural killer (NK) cells and neutrophils. It also exhibits antiproliferative activity against some cells. Recombinant IFNγ is a therapeutic agent that is similar to the endogenous IFNγ and exhibits immunoregulatory, antiviral, and antineoplastic activities.[28] It is FDA approved for the treatment of chronic granulomatous disease[29] and has also been used in atopic dermatitis[30] and cutaneous T cell lymphoma.[15] The contraindications and adverse effects are similar to those of IFNα.[29]

### Interleukin 1 receptor antagonist (IL1Ra, Anakinra)

Anakinra is the non-glycosylated form of human IL-1Ra and is produced by recombinant DNA technology. Unlike the naturally occurring IL1Ra there is an additional methionine residue at the amino terminus. It acts as a biologic response modifier, by blocking the functions of the naturally occurring IL-1, including inflammation. It is indicated in rheumatoid arthritis.[31] Good results have also been reported in Schnitzler's syndrome,[32] familial cold autoinflammatory syndrome[33] and psoriatic arthropathy.[34] It is given by subcutaneous injection 100 mg once a day. It has been co-administered with good effect, with methotrexate, in rheumatoid arthritis[35] and with thalidomide in Schnitzler's syndrome.[36] However, due to the dangers of drug interaction, with increased risk of neutropenia and serious infections, it is contraindicated with TNFα blockers (Infliximab, Etanercept, and Adalimumab). It is also contraindicated in patients with hypersensitivity to Anakinra, pre-existing malignant diseases, neutropenia, impaired renal function, pre-existing active tuberculosis, lactating mothers, and pediatric patients under age 18. It should be given with caution to women of child-bearing age, pregnant women, geriatric patients, and patients with asthma. The side effects that may occur with Anakinra include ecchymosis, fungal infections, LE-like syndrome, urticaria. Melanoma, interstitial granulomatous drug reactions (rarely), nausea, diarrhoea, abdominal pain, infections, flu-like symptoms, neutropenia, arthritic symptoms, injection site pain, inflammation and erythema, and rarely anaphylactic reactions.[37] In recent times, new onset of psoriasis following treatment with Anakinra has been reported.[38]

### Interleukin 2

IL-2 is produced by Th1 cells activated by antigen presenting cells (APC). IL-2 acts on activated T and B cells, NK cells, and macrophages, to stimulate the growth and differentiation of T cells and activation of NK cells. Recombinant IL-2 is an antitumor cytokine that has been used in cutaneous T cell lymphoma (CTCL) and metastatic melanoma.[39] When given intravenously in high doses of 600,000-720,000 IU/kg in melanoma, IL-2 has produced a 15-20% overall response, with complete cure in 4-6%.[40] It is a pregnancy category C drug, which is contraindicated in patients who are hypersensitive to IL-2 and those who have a history of abnormal thallium stress test, abnormal pulmonary function tests, and organ allograft transplantation.[41] The side effects include fever, chills, hypotension, pulmonary edema, thrombocytopenia, vascular leak syndrome, cardiac arrhythmias and exfoliative dermatitis.[42]

### Interleukin 4 (rhIL-4)

IL-4 is produced by activated Th2 cells, activated NK cells, mast cells, and basophils, and causes selective differentiation of Th2 cells.[43] In a dose-escalation study (0.5 to 5mg/kg is given by subcutaneous injection thrice a week) IL-4 has been shown to cause improvement in psoriasis by inducing Th2 differentiation in human CD4^+^ T cells.[44]

### Interleukin 10 (rhIL-10, Tenovil)

IL-10 is produced by Th2 cells, keratinocytes, mast cells, macrophages, and a subset of B cells. IL-10 is regarded as an anti-inflammatory cytokine because it acts on antigen presenting cells such as monocytes, macrophages, and dendritic cells, causing a decrease in the production of cytokines such as IL-1, IL-6, and IL-12. It also acts on Th1 cells to inhibit production of Th1 cytokines such as IL-2, TNFβ, and TNFγ. It stimulates B cells and causes activation of Th2 cells.[45] It is underexpressed in psoriatic lesions. rhIL-10 has been shown to improve psoriasis at doses of 4 mg/kg by subcutaneous injections and to produce long-term disease remission in various studies.[46]

### Interleukin 11 (rhIL-11, Oprelvekin)

IL-11 interacts with a variety of hemopoietic and nonhemopoietic cell types — interaction with macrophages leads to decreased production of type1 cytokines and interaction with T cells leads to differentiation along the Th1 lineage. It exhibits anti-inflammatory properties.[47] It is FDA approved for the treatment of chemotherapy—induced thrombocytopenia and has also shown reasonably good results in the treatment of psoriasis at doses of 2.5 or 5mg/ kg, by subcutaneous injection.[48]

### GM-CSF

GM-CSF is secreted by macrophages, T cells, mast cells, endothelial cells, and fibroblasts. It acts a white blood cell growth factor by stimulating stem cells to produce granulocytes and monocytes, which migrate into tissues and mature into macrophages.[49] It also induces proliferation and differentiation of adenomatosis polyposis coli.[50] Recombinant human GM-CSF has been used in dermatology to promote wound healing in ulcerated skin[51] (e.g., leg ulcers)[52] and for the treatment of melanoma[53] and Sezary syndrome.[54]

### PDGF

PDGF is produced by platelets, neutrophils, macrophages, and smooth muscle cells. It is a dimeric glycoprotein composed of two A or two B chains. The homodimer PDGF-BB regulates cell growth and division and promotes granulation tissue formation, re-epithelialization and wound angiogenesis.[55] Recombinant PDGF-BB topical gel (100μg/g), applied once daily, has been approved by FDA for the treatment of diabetic foot ulcers.[56,57]

## Monoclonal Antibodies

Monoclonal antibodies target specific cell-surface receptors. In the early days of biologic therapy, purely murine monoclonal antibodies were used. However, due to the development of antimurine antibodies, which blocked their action, these could be given only for very short periods. The monoclonal antibodies used now have genes that have different amounts of murine sequences in the variable region. At present the monoclonal antibodies may be categorized into three classes (a) chimeric antibodies comprising of 30% murine genes fused with human antibodies (b) humanized antibodies, which have 10% murine sequences, and (c) human antibodies, which are solely derived from human immunoglobulin genes.[58] The principal monoclonal antibodies with therapeutic relevance in dermatology are:

- Anti-TNFα: Infliximab, Adalimumab, Certolizumab, Golimumab
- Anti-LFA1: Efalizumab
- Anti-CD20: Rituximab
- Anti-IL-12 and anti-IL-23 monoclonal antibody: Ustekinumab
- Anti-CD2 antibody: Siplizumab
- Anti-CD4 antibody: Orthoclone (OKTcdr4a)
- Anti-CD25 antibodies: Basiliximab, Daclizumab
- Anti-CD80r: Galiximab (IDEC 114)
- Anti-IgE: Omalizumab

### Anti TNFα: Infliximab, adalimumab, certolizumab, golimumab

#### Infliximab:

It is a 149kd chimeric human mouse monoclonal antibody, consisting of a murine antigen-binding region, with specificity for TNFα fused to the human IgG1 constant region. It binds to the soluble and transmembrane forms of TNFα and triggers complement-mediated lysis of cells producing TNFα, thus reducing cutaneous inflammation and promoting keratinocyte differentiation. It also induces apoptosis of lesional keratinocytes. It is distinguished from other TNFα inhibitors by its ability to fix, complement, and lyse target cells.[59] Infliximab is indicated in psoriasis vulgaris, psoriatic arthropathy, pustular psoriasis, sarcoidosis, Crohn's disease, ulcerative colitis, rheumatoid arthritis, and ankylosing spondylitis.[60,61] It has also been reported to be useful in hidradenitis suppurativa, subcorneal pustular dermatosis, pyoderma gangrenosum, toxic epidermal necrolysis, Behçet's syndrome, and SAPHO syndrome. Depending on the indication the dosage varies from 3-10mg/kg (as IV infusion, given over several hours at weeks 0, 2 and 6 and every 8 weeks thereafter).[6] The most serious side-effect reported is the risk of infections particularly tuberculosis[62] and deep fungal infections such as histoplasmosis, cocidioiodomycosis, and blastomycosis.[63] Other infections reported are pneumonia, abscess, cellulitis, skin ulcers, and pyelonephritis. The risk of opportunistic infections has been found to be higher in the first year of treatment.[6] Infusion reactions that occur in 10% of the patients who develop antibodies to Infliximab may be immediate (anaphylactic reaction – convulsions, hypotension, and erythematous rash) or delayed (myalgia, arthralgia, fever, pruritus, edema of face/hands/lip, urticaria, rash, dysphagia, sore throat, headache, vertigo, or flushing). Such reactions can be avoided by premedicating with antihistamines, slowing down the infusions, and treating with systemic corticosteroids.[64] Other side effects include congestive heart failure (risk of worsening of heart failure in patients on high dose), demyelinating disease, and development/recurrence of lymphoma or other malignancies.[6]

#### Adalimumab:

It is a human IgG1 monoclonal antibody, which binds soluble and transmembrane TNFα, blocks the interaction of TNFα with p55 and p75 cell surface receptors, lyses cells that express TNFα in the presence of a complement, and increases the number of epidermal Langerhans cells in psoriatic plaques.[59] It is indicated for the treatment of rheumatoid arthritis, psoriatic arthritis, ankylosing spondylitis, Crohn's disease, moderate-to- severe chronic psoriasis, and juvenile idiopathic arthritis.[42] Recently released data, from large, randomized clinical trials suggests that adalimumab is effective, safe, and appropriate for long-term use, indicating a beneficial effect on the quality of life parameters for the treatment of chronic plaque psoriasis, and is well tolerated. Thus, adalimumab seems to be a promising therapeutic approach for patients who suffer from moderate-to-severe plaque psoriasis.[65] Side effects that may occur with adalimumab include injection site reactions,[66] fatal blood disorders, tuberculosis, infections caused by viruses, fungi or bacteria, rarely lymphoma and solid tissue cancers, serious liver injury, demyelinating CNS disorders, and cardiac failure.[67]

#### Certolizumab pegol and golimumab:

Apart from the three TNF antagonists infliximab, adalimumab, and etanercept currently available, two other TNF antagonists, certolizumab and golimumab, are in various stages of clinical development.[68] Certolizumab is the recombinant antibody Fab' fragment of a humanized TNF inhibitor monoclonal antibody. It has been approved by FDA in 2008, for the treatment of Crohn's disease. A phase II, randomized, double-blind, placebo-controlled, dose-ranging study of 176 patients with moderate-to-severe chronic plaque psoriasis showed that both the 200mg and 400mg liquid formulations of certolizumab pegol, given subcutaneously every two weeks, over a period of 12 weeks, significantly reduces the redness, thickness, and scaliness of lesions compared to the placebo. Side effects reported were serious infections, particularly tuberculosis and malignancies.[69] Golimumab, a fully human monoclonal antibody is at present undergoing Phase III clinical trials in psoriatic arthropathy.[70]

### Efalizumab (Anti-LFA1)

Efalizumab is a 148kd humanized IgG1 monoclonal antibody directed against the CD11a subunit in LFA-1 (Lymphocyte function-associated antigen-1), which is a heterodimer of CD11a and CD18, and a member of the β2-integrin family of cellular adhesion molecules found on T cells, B cells, macrophages, and neutrophils. It interacts with ICAM-1 on APCs, endothelial cells, and keratinocytes, functions as an adhesion molecule and regulates T cell trafficking. CD11a is a more selective target for immunosuppression than CD18. Binding of efalizumab and LFA-1 blocks the interaction of LFA-1 and ICAM-1. Consequently LFA-1 on memory T cells is increased and ICAM-1 is expressed on endothelial cells at the site of inflammation and keratinocytes. Thus, efalizumab prevents binding of T cells to endothelial cells and blocks T cell movement into the skin without depleting the memory T cells.[71] Although efalizumab has been approved by FDA in October 2003, for moderate-to-severe plaque psoriasis, it has not been found to be effective in psoriatic arthropathy. It is injected subcutaneously with an initial loading dose of 0.7mg/kg followed by 1.0mg/kg weekly. The maximum dose should not exceed 200mg weekly.[72] Apart from its usefulness in plaque psoriasis, it is also considered as a first-line choice for hand and foot psoriasis, overweight patients, and those who have had inadequate response to a previous therapy with a TNF inhibitor.[73] Efalizumab has also been found to be useful in atopic dermatitis,[74] dermatomyositis,[75] discoid lupus erythematosus,[76] and lichen planus.[77] Adverse effects associated with efalizumab therapy included flu-like symptoms after the first two doses (rare after the third dose),[78] thrombocytopenia, and eczematous dermatitis.[79] Efalizumab is associated with a rebound flare reaction in approximately 5% of the patients after stopping the therapy.[80] It is contraindicated in patients with hypersensitivity to the drug, and patients on immunosuppressive drugs or vaccines. It is a pregnancy category C drug. No embryotoxicity or teratogenecity has been reported. Caution should be exercised when prescribing it to the elderly.[78]

### Rituximab (Anti-CD 20)

Rituximab is a humanized monoclonal antibody against CD 20, which is a cell surface protein expressed by mature and pre-B cells. It enhances antibody and complement-mediated cytotoxicity and promotes apoptosis of malignant CD20^+^ cells.[81] It is indicated for use in moderate-to-severe rheumatoid arthritis, CD20^+^ non-Hodgkin B cell lymphoma (both FDA approved), paraneoplastic pemphigus, urticarial vasculitis, SLE, cutaneous B cell lymphoma, epidermolysis bullosa aquisita, and recalcitrant pemphigus. The recommended dosage is 375mg/m^2^ body surface area by intravenous infusions at weekly intervals of 4-8 doses. Complete blood counts and serum antibody levels should be monitored every two months. Adverse effects associated with rituximab therapy are infusion reactions (avoided by giving acetaminophen and antihistaminic premedication), hepatitis B reactivation, renal toxicity, tumor lysis syndrome, progression of Kaposi's sarcoma, mucocutaneous reactions, bowel obstruction/perforation.[42]

## Ustekinumab (Anti IL-12 And Anti IL-23)

It is a fully human monoclonal antibody targeting IL-12 and IL-23, presently undergoing clinical trials for psoriasis and psoriatic arthropathy.[2,82] In a phase III, double- blind, placebo- controlled study, 766 patients with moderate- to- severe psoriasis (PHOENIX 1) were given ustekinumab 45mg or 90mg subcutaneously at weeks 0 and 4 and then every 12 weeks. It was found that ustekinumab given every 12 weeks provided sustained, clinically meaningful improvement in the treatment of moderate-to-severe plaque psoriasis through one year.[83] In another study (PHOENIX 2) 70% patients with moderate-to-severe plaque psoriasis, receiving two subcutaneous doses of ustekinumab, had a 75% reduction in psoriasis at week 12.[84] Both studies showed that ustekinumab could control plaque psoriasis with only four injections a year resulting in greater ease of use and more sustained relief.

## Siplizumab or Medi-507 (Anti-CD2)

It is a humanized IgG1 monoclonal directed against CD2. CD2 is expressed on the surface of both CD4^+^ and CD8^+^ memory T cells (CD45RO^+^), T cells, and NK cells. The blocking of CD2 selectively inhibits the activation and proliferation of the pathogenic memory T cells, thus providing relief in psoriasis. [85] In an open label, dose-escalation study, 39 patients with moderate-to-severe psoriasis were injected with 0.1-0.7mg weekly for 12 weeks. Although reduction in Psoriasis Area and Severity Index (PASI) scores was observed at all doses, it was more pronounced at higher doses.[86]

## Orthoclone or OKTcdr4a (Anti-CD4)

It is a humanized antihuman CD4 IgG4 monoclonal antibody derived from the murine OKT4A. It prevents the recognition of the MHC-bound antigen by an appropriate T-cell receptor, hence the T cells do not get activated.[87] Several studies have found orthoclone to be effective in moderate-to-severe psoriasis.[88,89]

## Basiliximab (Anti-CD 25)

Basiliximab is a chimeric mouse-human monoclonal antibody directed against the IL- 2Rα receptor (also known as the CD25 antigen) of T cells. It has been successfully tried in severe recalcitrant psoriasis and palmoplantar pustular psoriasis both in combination with cyclosporine[90] as well as a cyclosporine sparing agent.[91]

## Daclizumab (Anti CD-25)

It is a humanized monoclonal antibody directed against CD25 (α-subunit). It has been currently evaluated for the treatment of psoriasis with moderately good results.[92]

## Galiximab (IDEC – 114, Anti-CD80)

It is a primatized monoclonal antibody with a human IgG1 constant region. It binds to the CD80 receptor found on APCs and certain activated T cells. CD80 is a costimulatory molecule involved in T cell activation. By binding to CD80, galiximab causes a reduction in lesional, activated T cells, causing improvement in the psoriatic plaques.[93] In open-label studies it has been found that a single dose of galaximab is safe and well-tolerated, with preliminary evidence of clinical and histological response in Psoriasis.[94,95]

## Omalizumab (Anti-IgE)

It is a recombinant, humanized, monoclonal antibody against immunoglobulin IgE. It binds IgE at the same site as its high-affinity receptor, FcεR I causing reduced binding of IgE to FcεR I. Consequently IgE is prevented from sensitizing cells bearing high-affinity FcεR I receptors resulting in a rapid depletion of both cell- bound IgE and surface FcεR I expression on blood basophils. Omalizumab has been tried with good results in atopic dermatitis[96] and chronic urticaria.[97] It has been found to be a particularly promising therapeutic option for antihistamine unresponsive chronic autoimmune urticaria.[98]

## Fusion Antibody Proteins

Fusion proteins, also known as chimeric proteins, are proteins which are created by the fusion of the receptor domain of a human protein with the constant region of human IgG. The resultant fusion protein binds specifically to a ligand or co-receptor. Recombinant fusion proteins have also been produced by combining human proteins with bacterial toxins.[99] The fusion proteins most commonly used in dermatology are:

- Etanercept
- Alefacept
- Abatacept
- Denileukin Diftitox

### Etanercept

It is a fully human dimeric fusion protein formed by the combination of two identical TNFα type II (p75) receptor peptides, which are fused to the Fc portion of the human IgG1. It binds both TNFα and TNFβ in circulation. Consequently both the molecules are prevented from activating their cell surface receptors TNF-R1 and TNF-R2. It acts in psoriasis by decreasing the epidermal thickness and by decreasing NFκB transcriptional activity in psoriatic skin. It also reduces IL-8, IL-17, IL-22, and MIP-3a (CCL20) expression in psoriatic skin causing a decrease in T cells and dendritic cells, and a reduction in keratinocyte hyperplasia.[59] It is FDA approved for the treatment of rheumatoid arthritis, ankylosing spondylitis, psoriatic arthropathy, and plaque psoriasis (moderate-to-severe). It is also effective in scleroderma, recurrent aphthous ulcers, and patients of immunodeficiency, presenting with scarring alopecia, arthritis, diarrhea, and recurrent infections.[42] Self-administered subcutaneously, once or twice weekly, it is given in plaque psoriasis at doses of 50 mg/kg/week, twice weekly for three months, with a maintenance dose of 50 mg twice weekly in adults and doses of 0.8mg/kg/week in pediatric patients. In psoriatic arthritis and rheumatoid arthritis it is administered in doses of 25mg twice weekly.[42,100] The injections are given on the abdomen, thighs, or upper arms, at one-inch distance from the previous injections or at contralateral sites.[6] Many patients with moderate-to-severe plaque psoriasis do not respond adequately to methotrexate monotherapy. Addition of etanercept to methotrexate achieves significant improvement in psoriasis after 24 weeks.[101] The adverse effects of etanercept are similar to those seen with infliximab, and include infections, particularly reactivation of latent tuberculosis, injection site reactions, headache, rhinitis, malignancies, congestive heart failure, and demyelinating disease.[67] In addition, drug-induced systemic lupus erythematosus and subacute cutaneous lupus eryhtematosus have been reported.[102] Etanercept is contraindicated in patients with hypersensitivity to the drug and patients with active or chronic localized infections. Caution should be exercised in diabetes and CNS demyelinating disorders.[6]

### Alefacept

Alefacept is a recombinant fusion protein produced by the fusion of the first extracellular domain of LFA3 with hinge Ch1 and Ch2 sequences of human IgG1p. It is produced by recombinant DNA technology in the Chinese hamster ovary mammalian cell expression system. LFA3 is a member of the IgG superfamily expressed on APC. It is a ligand for CD2, which is expressed on T cells and NK cell-ligations of CD2 by LFA3, which is a signal for T cell proliferation and activation.[103] Alefacept blocks the LFA3-CD2 interaction and thus prevents the activation of T cells. Patients of psoriasis treated with alefacept show reduced activation of T cells and memory T cells.[104] Alefacept was the first biologic to receive FDA approval for the treatment of psoriasis in January 2003. In moderate-to-severe psoriasis it is given in doses of 15mg IM/7.5mg IV weekly for 12 weeks followed by a 12-week holiday. Improved efficacy has been observed if the drug is given for 16 weeks. It can be given safely for up to five courses. CD4 count has to be completed before starting treatment and should be monitored every two weeks during therapy. If the CD count falls below 250 then treatment is discontinued till the count rises again. However, if the CD count is persistently below 250 for four consecutive weeks, then the drug has to be discontinued permanently.[105,106] The efficacy of alefacept has been found to be increased by combining alefacept with short-term (6-12 weeks) UVB treatment.[107] It has been found to be useful in the treatment of psoriatic arthropathy either alone[108] or in combination with methotrxate.[109] Some of the off label conditions where alefacept has been used with success are *graft-versus-host disease* (GVHD), lichen planus, alopecia areata,[42] atopic dermatitis,[110] mycosis fungoides,[111] alopecia universalis,[112] erosive lichen planus,[113] Hailey-Hailey disease,[114] and hand dermatitis. [115] It is contraindicated in patients with hypersensitivity to the drug, in pediatric patients, in HIV infections, and if the CD4 count is below normal. It is a pregnancy category B drug, its safety in lactation is unknown. It should be used with caution in elderly patients.[42] Although no increased incidence of infections have been reported alefacept should be avoided in patients with pre-existing infections or those on immunosuppressive drugs. Reported side effects include transient chills, injection site reactions, and a few cases of malignancies including lymphomas reported during trials.[6]

### Abatacept (CTLA4Ig)

It is a fusion protein composed of the extracellular domain of CTLA4 and the Fc region of IgG4. It interferes with T-cell activation by competitively binding the B7.1 and B7.2 molecules on the surface of APC.[116] In an open label, dose-escalation, multi-center study, 43 patients with stable psoriasis vulgaris were divided into eight groups and given IV infusions of 0.5, 1, 2, 4, 8, 16, 25, and 50mg/kg of CTLA 4Ig on days 1, 2, 16, and 29. By week 25, 46% of the patients evaluated achieved 50% or more improvement.[117] It has been hypothesized that since abatacept suppresses T-cell function, it has the potential to be a treatment choice for psoriasis, where pathologic processes are driven by T cells.[118] A second generation CTLA4Ig, Belatacept, is currently under Phase II Clinical trial for allograft diseases.[119]

### Denileukin diftitox

DAB389IL-2 (Denileukin diftitox) is a recombinant fusion toxin formed by linking of human IL-2gene and the enzymatically active ADP-ribosyltransferase domain of the diphtheria toxin. It binds to the IL-2 receptor on the cell membrane and is then internalized within the endosome via receptor-mediated endocytosis. Subsequently the ADP-ribosyltransferase unit is cleaved and translocated to the cytoplasm causing interruption in protein translation and inducing cell apoptosis.[120] It is approved by the FDA for the treatment of *cutaneous T-cell lymphoma* (CTCL) at doses of 9μg/kg/day or 18μg/kg/day IV over 15minutes.[121] It has also been evaluated in psoriasis with moderately good results.[122] The side effects that may occur are hypersensitivity reactions (comprising of fever, hypotension, dyspnea, arthralgias, rash, and tightness of the chest/back), flu-like symptoms, reversible transaminitis, vascular leak syndrome, and morbilliform rash. It is a pregnancy category C drug and the safety in lactation is unknown. It is contraindicated in patients who are hypersensitive to the drug.[42]

## Conclusion

Biologics represent the future of therapeutics, not only in dermatology but also in other fields of medicine. Among all the dermatological disorders it is psoriasis where the biologics have been most evaluated.[123] Vastly different opinions have been expressed about the comparative efficacy of the various biological therapies available for psoriasis. Based on a review of the literature available from 1986 to 2006, Leon *et al.,* opined that the percentage of PASI 75 reduction at approximately 12 weeks, obtained by different biologics were; infliximab, 80%; adalimumab 40mg every other week, 53%, and 40mg/week, 80%; etanercept 50mg twice weekly, 49% and 25mg twice weekly, 34%; efalizumab, 31.4%; and alefacept 21%.[124] Brimhall *et al.*, opined that considering the efficacy as well as safety of biologics, the most favorable drug was infliximab followed by etanercept, efalizumab, and alefacept (adalimumab was not considered).[125] According to Schmitt *et al.*, infliximab was the most efficacious followed by adalimumab.[125] As mentioned earlier various combination therapies have been proposed to increase the efficacy of the drugs.[126] However, the possibility of serious infections and the oncogenic potential, combined with the high cost of drugs, limit their use at the present stage.[42,127]
